# Supplementary material for: Determining Critical Thresholds of Environmental Flow Restoration Based on Planktonic Index of Biotic Integrity (P−IBI): A Case Study in the Typical Tributaries of Poyang Lake
Source: Int J Environ Res Public Health. 2022 Dec 22;20(1):169. doi: 10.3390/ijerph20010169 (PMC9820035; doi:10.3390/ijerph20010169)
Supplement: Supplementary file 1 [file ijerph-20-00169-s001.zip › ijerph-2073505-supplementary.pdf]

Supplementary materials

Table S1 Hydrological station data duration

Hydrological station	duration	Number of data years
SZL	1957 - 2018	62
ST	1974 - 2015	42
XX	1970 - 2015	46
GX	1970 - 2018	49
TP	1970 - 2018	49
LOU	1953 - 2018	66
LIA	1953 - 2018	66
LID	1956 - 2019	64

Table S2 Monthly average flow difference

Hydrological station	SZL	LOU	LID	LIA	ST	XX	GX	TP
1	2.4	24.05	74.7	86	4.2	0.464	3.77	0.853
2	10.3	73.7	254.5	188	18.3	1.342	10.44	1.347
3	13.35	47.8	109	80	14.9	0.68	6.35	1.828
4	-0.25	20.7	13	59	10.8	0.51	-2.85	2.162
5	11.75	2	102	129	12.7	0.04	-0.15	1.27
6	-20.65	-103	-443	-378	-34.3	-1.21	-13.38	-2.3
7	-4.1	-45.05	-114.1	-156	-9.2	-0.86	-1.98	-2.7
8	-4.85	-6.27	-21.25	-45.35	-7.4	-0.42	-1.595	-0.915
9	-4.35	-19.48	-10.45	-24.75	-5.7	-0.4185	-0.725	-0.39
10	-3	-0.77	34.9	49.2	-1.6	-0.0905	0.44	-0.975
11	-2.1	3.77	5.8	12.9	-1.6	-0.048	-0.18	-0.415
12	-1.5	-2.55	5.1	0	1.1	-0.011	0.14	-0.235
